# Quantitative evaluation of retinal vascular parameters among highland and lowland native children using artificial intelligence

**DOI:** 10.3389/fcell.2025.1693739

**Published:** 2025-10-15

**Authors:** Xueqing Bai, Chengyue Zhang, Xingye Wang, Huizhen Tan, Yinuo Wang, Alimujiang Abula, Tianqi Lan, Yuxiang Liao, Xiaofang Ju, Xiaojie Quan, Xue Han, Xue Zhang, Li Li

**Affiliations:** ^1^ Beijing Children’s Hospital Capital Medical University, Ophthalmology, Key Laboratory of Major Diseases in Children, Ministry of Education, Beijing, China; ^2^ Children’s Hospital of Xinjiang Uygur Autonomous Region, Ophthalmology, Urumqi, Xinjiang, China; ^3^ EVision Technology (Beijing) Co. Ltd., Beijing, China; ^4^ Qinghai Provincial Women and Children’s Hospital, Ophthalmology, Xining, Qinghai, China; ^5^ Imperial College London, Exhibition Rd, South Kensington, MSci Chemistry with Medicinal Chemistry, London, United Kingdom

**Keywords:** retinal vasculature, optic nerve head, highland, children, AI

## Abstract

**Introduction:**

To investigate retinal vascular and optic nerve head (ONH) characteristics in highland- and lowland-dwelling native children using artificial intelligence-based automated quantification of retinal vascular morphology.

**Methods:**

This cross-sectional study analyzed 834 fundus images from 417 children (age 8–9 years), including 123 highland children (HLC) and 294 lowland children (LLC). Fundus photography was performed using a non-mydriatic 45° fundus camera. Automated image analysis quantified: (1) retinal vascular parameters (fractal dimension [VDf], caliber [VC], tortuosity [VT], density [VD], branching angle [VBA], and arteriole-to-venule ratio [AVR]); and (2) optic nerve head morphology (disc area, cup area, and cup-to-disc ratio [C/D]). Group comparisons of all parameters were conducted using AI-based analytical methods.

**Results:**

After adjusting for age, sex, and axial length (AXL), covariance analysis demonstrated that the HLC group showed significantly smaller VC (p = 0.005) and VBA (p = 0.003), along with larger optic cup and disc areas (p < 0.001) compared to the LLC group. Both peripapillary VD and VC exhibited a progressive decrease with increasing distance from the optic disc border. Moreover, the HLC group demonstrated a significantly lower VC compared to the LLC group (p = 0.005). Furthermore, the HLC group displayed: (1) significantly higher VD in the inferior quadrant (p < 0.001), (2) increased VT in both nasal and temporal quadrants (p < 0.001 for both), (3) decreased VC in superior (p < 0.001), inferior (p = 0.005), and nasal (p = 0.001) quadrants, and (4) reduced VDf in the nasal quadrant (p = 0.001) of peripapillary regions compared to the LLC group.

**Conclusion:**

Our findings demonstrate statistically significant differences in retinal vascular and optic nerve head morphology between HLC and LLC. Specifically, the HLC group exhibited significantly reduced VC but larger optic cup and disc areas compared to the LLC group.

## Introduction

High-altitude environments are defined as geographical regions situated at elevations typically above 2,400 meters (m) above sea level (Stacey et al., 2023). At these elevations, the decreased atmospheric pressure leads to several characteristic environmental challenges: (1) reduced oxygen partial pressure (hypoxia), (2) decreased air density, (3) lower ambient temperatures, and (4) increased weather variability compared to lowland areas. Notably, physiological adaptation becomes progressively more demanding, with each 500-m elevation gain above 3,500 m significantly impacting human physiology and acclimatization processes (West, 1984).

Prolonged hypoxia induces significant hemodynamic alterations in both cerebral and ocular circulation (Frayser et al., 1974). Physiological adaptations to chronic hypoxic exposure include attenuated hypoxic pulmonary vasoconstriction, increased hemoglobin concentration, and expanded plasma volume - critical compensatory mechanisms that enhance oxygen delivery (Bartsch and Gibbs, 2007). Cerebral blood flow demonstrates a characteristic biphasic response to high-altitude exposure: an initial increase during the first 24 h, followed by gradual normalization over subsequent days (Dyer et al., 2008), ultimately stabilizing following complete acclimatization (Moller et al., 2002).

Remarkably, despite these environmental challenges, approximately 140 million people have permanently settled in high-altitude regions, including the Andean, East African, and Tibetan plateaus, demonstrating exceptional physiological adaptations shaped by long-term hypobaric hypoxia and evolutionary selection (Beall, 2006; Mishra et al., 2015; Simonson, 2015).

Prolonged hypoxia induces blood flow changes in both cerebral and ocular circulations (Delaey and Van De Voorde, 2000; Patton et al., 2005). Retinal blood vessels are the only directly observed blood vessels in the body; changes in them can help effective assess the occurrence and development of systemic and ocular diseases (Xu and Yang, 2023; Chen et al., 2024). During hypoxic exposure, retinal vessels undergo characteristic dilation and increased tortuosity to meet elevated metabolic demands (Neumann et al., 2016). In recent years, advancements in retinal imaging technology and artificial intelligence (AI) algorithms have enhanced the specificity and efficiency of quantitative assessment techniques for retinal vascular changes, leading to their widespread application (Xu and Yang, 2023). In this study, we employed these techniques to quantitatively evaluate the effects of prolonged low-pressure hypoxia on retinal vascular development in children at high altitude.

The Qinghai-Tibetan Plateau, with an average elevation of 4,000 m, represents one of the world’s highest inhabited regions, encompassing the Yushu Tibetan Autonomous Prefecture. Extensive evidence indicates that indigenous high-altitude populations have evolved unique physiological adaptations to chronic hypobaric hypoxia (Jeong et al., 2018). This study investigates the long-term effects of hypoxemia on retinal hemodynamics by comparing high-altitude natives with lowland populations. We hypothesize that chronic high-altitude exposure induces distinct retinal vascular adaptations, particularly given the current paucity of research examining these characteristics in pediatric populations residing at extreme elevations.

## Methods

### Participants

This cross-sectional study examined two distinct altitude regions in western China: the high-altitude Yushu Tibetan Autonomous Prefecture (4,000 m above sea level) and the low-altitude Shihezi area (450 m above sea level). Yushu, situated in southeastern Qinghai Province, has a predominantly Tibetan population (98%), while Shihezi, located in central Xinjiang Uygur Autonomous Region, is predominantly Han Chinese (94%).

To minimize age-related confounding factors, we recruited native children aged 8–9 years from local schools in both regions. Participants were categorized into two groups: the highland children (HLC) group (*n* = 123 Tibetan children from Yushu) and the lowland children (LLC) group (*n* = 294 Han children from Shihezi). The HLC and LLC groups were composed of children who had resided exclusively at their native altitudes (≥4,000 m for HLC and ≤500 m for LLC) since birth. Migrants and individuals with chronic illnesses were excluded.

All participants underwent comprehensive ophthalmic evaluations, including: best-corrected visual acuity (BCVA), intraocular pressure (IOP), slit-lamp, fundus, autorefractor NIDEK (ARK-700A; NIDEK; JAPAN) and ocular biometry (StarEyes900, Wanling Bang Bridge, China).

The inclusion criteria included the following:

1. 8–9 years old;
2. BCVA ≥20/25;
3. Spherical equivalent (SE) ≤ 0.5D
4. IOP ≤21 mmHg;
The exclusion criteria included the following:
1. Axial length (AXL) ≥ 26.0 mm;
2. History of excimer laser surgery, intraocular surgery or ocular injury;
3. Subjects with mental illness precluding examination cooperation or any ocular/systemic conditions potentially affecting retinal vasculature.

### Fundus photographs

After sitting in the darkened room for approximately 5 min to allow for natural pupil dilation, digital fundus photography was performed by trained technicians using a non-mydriatic handheld fundus camera (RetinaVue 100, Welch Allyn). The device captured 45-degree color fundus images centered on the optic disc, ensuring the resulting photographs were suitable for artificial intelligence analysis.

### AI-enabled retinal image processing and quantification of parameters

**FIGURE 1:**
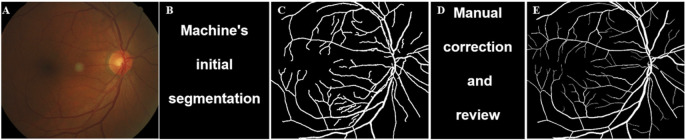
Vascular labeling process of fundus image. The specific process includes: **(A)** Original image; **(B)** Machine’s initial segmentation; **(C)** Preselected vascular regions; **(D)** Manual correction and review; **(E)** Final labeled sample.

**FIGURE 2:**
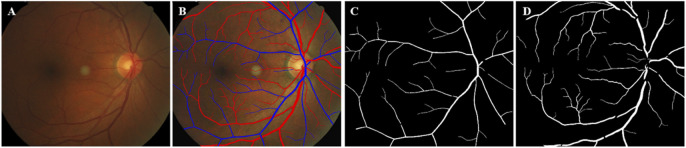
Schematic diagram of vascular segmentation. **(A)** Original; **(B)** Vascular extraction effect diagram, red represents arteries and blue represents veins; **(C)** Segmentation map of retinal arteries; **(D)** Segmentation map of retinal veins.

**FIGURE 3:**
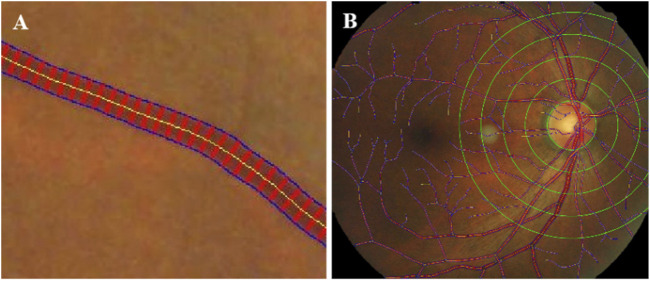
Schematic diagram of vessel diameter measurement. **(A)** Vascular diameter measurement, Yellow line was the centerline. Blue lines showed vessel boundary. Red lines were orthogonal to the centerline. **(B)** Vascular diameter measurement by region, Zoning with 0. 5 PD as a reference distance.

**FIGURE 4:**
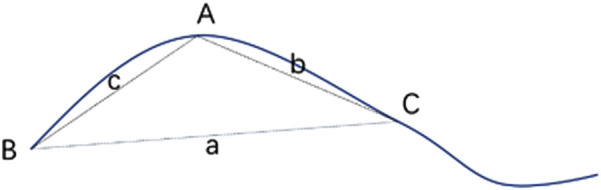
Schematic diagram of blood vessel curvature calculation.

**FIGURE 5:**
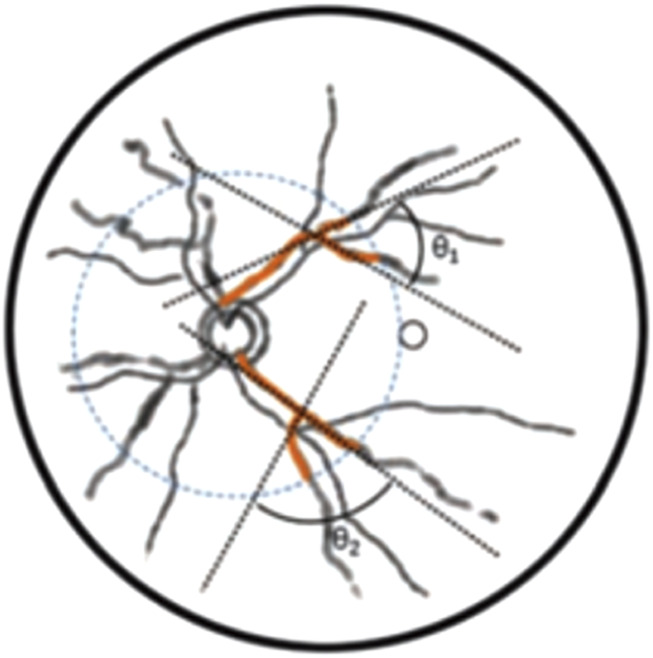
Schematic diagram of intersection angle of vascular branches. Blue dashed line was 2 PD range of optic disk, and black dashed line were fitted straight lines to points 10 pixels from vascular branches.

In this paper, the fundus image intelligent analysis software—EVisionAI is used to intelligently process the fundus image (Xu et al., 2019). The software is designed based on the bionic mechanism of human vision and deeply integrates image processing technologies such as computer vision and deep learning, which first performs Regions of Interest (ROI) extraction, denoising, normalization, and enhancement operations to remove invalid areas such as the image background and reduce image noise and image differences (Xu et al., 2019; Zhang et al., 2022). Then it combines the deep learning network model with the edge extraction algorithm based on the visual attention mechanism to finely identify and segment the features of the optic disc, and retinal blood vessels, respectively (Zhang et al., 2022). This study systematically presents several key technical components using an AI-based quantitative analysis approach, encompassing the vascular labeling workflow (Figure 1), the segmentation results (Figure 2), as well as quantitative parameterization indicators such as vascular diameter (Figure 3), tortuosity (Figure 4), and branch angle (Figure 5). Based on the segmentation results, the retinal vascular and optic nerve head (ONH) parameters, such as vascular fractal dimension (VDf), vascular caliber (VC), vascular tortuosity (VT), vascular density (VD), vascular branching angle (VBA), area of optic disc, arteriole-to-venule ratio (AVR), and cup-to-disc ratio (C/D), were measured. This comprehensive digital characterization aims to provide a detailed description of the color fundus photographs and their underlying structures. The computation of vascular fractal dimension (VDf) mainly involves the following steps: (1) retinal image preprocessing, (2) extraction of retinal blood vessels, and (3) calculation of the VDf. The accuracy of feature recognition for each result was ≥0.96, sensitivity ≥0.85, and specificity ≥0.96.

### Statistical analysis

All statistical analyses were conducted using SPSS software (version 20.0; IBM Corp.). Continuous variables are expressed as mean ± standard deviation (SD), while categorical variables are presented as frequencies and percentages (%). Data from both eyes were included in the analysis (Table 1). Demographic characteristics (age, gender) and ocular parameters (AXL) were compared between groups using independent samples t-tests for continuous variables and chi-square tests for categorical variables. Given the observed differences in age, sex distribution, and AXL between subgroups, these variables were included as covariates in subsequent analyses. Analysis of covariance (ANCOVA) was employed to evaluate group differences in retinal vascular and ONH parameters, with adjustment for the aforementioned covariates. A two-tailed p-value <0.05 was considered statistically significant for all analyses.

**TABLE 1:** Participant characteristics.

Children characteristics	HLC	LLC	p
Number (eyes)	123 (246)	292 (584)	
Age (years, Mean ± SD)	8.66 ± 0.66	8.08 ± 0.28	<0.001
Male, n (%)	60 (43.80%)	362 (52.20%)	<0.001
Axial length	22.76 ± 1.51	23.27 ± 0.79	<0.001

## Results

### Demographic characteristics

This study enrolled 417 school-aged children (8–9 years), consisting of 123 highland children (HLC; 29.6%) and 294 lowland children (LLC; 70.4%). As shown in Table 1, the two groups demonstrated significant differences in age distribution, gender composition, and AXL (all p < 0.001). To account for these baseline differences, we incorporated age, gender, and AXL as covariates in the ANCOVA model when comparing retinal vascular and ONH parameters between groups.

### Retinal vascular and ONH parameters in HLC and LLC groups

Following adjustment for age, gender, and AXL as covariates, statistically significant differences were observed between HLC and LLC in several ocular parameters (Table 2). The HLC group exhibited: larger optic disc area (2.549 mm^2^ vs. 2.365 mm^2^; p < 0.001); greater optic cup area (0.625 mm^2^ vs. 0.557 mm^2^; p < 0.001); smaller VBA (62.161° vs. 63.611°; p = 0.003); and reduced vascular caliber (77.865 μm vs. 81.663 μm; p = 0.005). In contrast, no statistically significant intergroup differences were observed in: VDf (p = 0.583), VT (p = 0.574), VD (p = 0.635), AVR (p = 0.055), and C/D (p = 0.101).

**TABLE 2:** Retinal vascular and ONH parameters in two groups.

Retinal vascular parameters	HLC (*n* = 123)		LLC (*n* = 294)		p
	Mean	SD	Mean	SD	
Vascular fractal dimension	1.490	0.082	1.486	0.063	0.583
Vascular tortuosity (×10^–3^)	0.888	0.116	0.898	0.121	0.574
Vascular density (%)	0.069	0.021	0.068	0.019	0.635
Vascular branching angle (°)	62.161	7.803	63.611	7.487	0.003
Vascular caliber (μm)	77.865	14.271	81.663	13.084	0.005
Arteriole to venule ratio	0.792	0.088	0.783	0.08	0.055
Area of optic cup (mm^2^)	0.625	0.252	0.557	0.227	<0.001
Area of optic disc (mm^2^)	2.549	0.476	2.365	0.4	<0.001
Cup to disc ratio	0.240	0.069	0.230	0.063	0.101

### Peripapillary retinal vascular parameters in HLC and LLC groups

**FIGURE 6:**
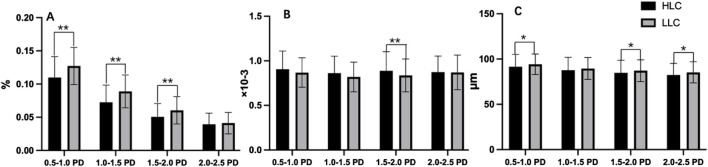
Comparisons of peripapillary vascular density (VD), vascular tortuosity (VT) and vascular caliber (VC) in the annular regions from the optic disc borders in highland children (HLC) and lowland children (LLC) groups. **(A)** VD. **(B)** VT. **(C)** VC (*p < 0.05, **p < 0.01).

Both peripapillary VD and VC showed reductions with increasing distance from the optic disc border. Furthermore, the HLC group exhibited lower VD and VC values compared to the LLC group (Figures 6A,C; Table 3). Notably, the HLC group demonstrated lower VD (p < 0.001) and VC (p = 0.026) compared to the LLC group within the 0.5–1.0 disc diameter (PD) zone. Regarding VT, the HLC group exhibited consistently higher values across all four annular zones, this difference reached statistical significance in the 1.5–2.0 PD region (p = 0.003) (Figure 6B; Table 3).

**TABLE 3:** Peripapillary retinal vascular parameters in two groups.

Retinal vascular parameters	HLC (*n* = 123)		LLC (*n* = 294)		p
	Mean	SD	Mean	SD	
Vascular caliber (μm)					
0.5–1.0 PD	91.51004702	16.303	94.2198428	12.582	0.026
1.0–1.5 PD	87.67174438	15.572	89.5910566	13.183	0.071
1.5–2.0 PD	84.76201121	16.098	87.1858475	13.828	0.033
2.0–2.5 PD	82.44703012	15.033	85.3621669	14.397	0.012
Vascular tortuosity (×10^–3^)					
0.5–1.0 PD	0.90615	0.203	0.86853	0.165	0.052
1.0–1.5 PD	0.86181	0.191	0.81903	0.167	0.045
1.5–2.0 PD	0.88828	0.214	0.83624	0.184	0.003
2.0–2.5 PD	0.87282	0.181	0.86999	0.194	0.709
Vascular density (%)					
0.5–1.0 PD	0.10984429	0.031	0.12727055	0.028	<0.001
1.0–1.5 PD	0.07251842	0.073	0.08897188	0.089	<0.001
1.5–2.0 PD	0.05052876	0.051	0.06045359	0.06	<0.001
2.0–2.5 PD	0.03935672	0.039	0.04116795	0.041	0.05

### Quadrant retinal vascular parameters in HLC group and LLC group

**FIGURE 7:**
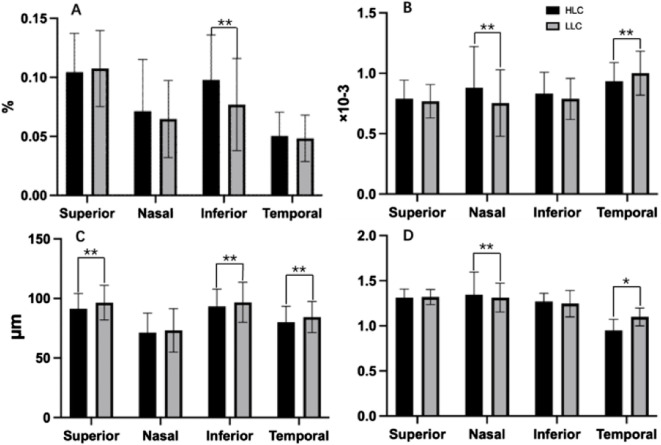
Comparisons of retinal vascular average density (VD), vascular tortuosity (VT), vascular caliber (VC) and vascular fractal dimension (VDf) in four quadrants of peripapillary areas in highland children (HLC) and lowland children (LLC) groups. **(A)** VD. **(B)** VT. **(C)** VC. **(D)** VDf (*p < 0.05, **p < 0.01).

Comparative analysis revealed significant regional variations in retinal vascular characteristics between groups (Table 4; Figure 7). The HLC group showed: greater VD in the inferior quadrant (0.098% vs. 0.077%, p < 0.001); increased VT in both nasal (0.934 × 10^−3^ vs. 0.754 × 10^−3^, p < 0.001) and temporal quadrants (0.881 × 10^−3^ vs. 0.754 × 10^−3^, p < 0.001); reduced VC in superior (91.323 μm vs. 96.409 μm, p < 0.001), inferior (93.266 μm vs. 96.732 μm, p = 0.005), and temporal quadrant (80.001 μm vs. 84.343 μm, p = 0.001); significantly lower VDf in nasal peripapillary regions (0.948 ± 0.032 vs. 1.099 ± 0.041; p < 0.001) but slightly higher VDf in temporal regions (1.345 ± 0.028 vs. 1.312 ± 0.035; p = 0.018) compared to LLC controls.

**TABLE 4:** Quadrant retinal vascular parameters in two groups.

Retinal vascular parameters	HLC (*n* = 123)		LLC (*n* = 294)		p
	Mean	SD	Mean	SD	
Vascular fractal dimension					
Superior	1.313	0.093	1.320	0.083	0.069
Nasal	0.948	0.251	1.099	0.16	<0.001
Inferior	1.270	0.092	1.246	0.147	0.099
Temporal	1.345	0.123	1.312	0.098	0.018
Vascular caliber (μm)					
Superior	91.323	14.531	96.409	12.436	<0.001
Nasal	71.310	18.297	73.193	16.431	0.132
Inferior	93.266	16.799	96.732	14.591	0.005
Temporal	80.001	13.039	84.343	13.453	0.001
Vascular tortuosity (×10^–3^)					
Superior	0.789	0.154	0.768	0.138	0.064
Nasal	0.934	0.341	0.754	0.276	<0.001
Inferior	0.831	0.169	0.788	0.177	0.139
Temporal	0.881	0.155	0.754	0.182	<0.001
Vascular density (%)					
Superior	0.105	0.033	0.108	0.032	0.19
Nasal	0.071	0.044	0.065	0.033	0.681
Inferior	0.098	0.038	0.077	0.039	<0.001
Temporal	0.05	0.02	0.048	0.02	0.83

## Discussion

This study employed artificial intelligence-based automated segmentation and quantitative analysis of retinal vasculature in color fundus photographs to systematically compare retinal vascular and ONH morphology between HLC and LLC groups. Our AI-based quantitative analysis revealed significant altitude-associated differences in multiple retinal parameters, including: VC, VBA, optic cup area, and optic disc area. To our knowledge, this represents the first systematic investigation demonstrating distinct retinal vascular patterns between highland and lowland pediatric populations using fully automated quantification methods.

Numerous studies have documented that retinal arterial and venous vessels undergo diameter increases under hypobaric hypoxia, with more prominent venous dilatation (Neumann et al., 2016; Yang et al., 2019). However, most existing research has involved low-altitude residents during brief high-altitude exposures, where acute mountain sickness (AMS) development correlated with reduced arterial constriction after ascending above 3,000 m (Westwood et al., 2024; Gupta et al., 2023). Atul et al. found that acute hypobaric hypoxia induces retinal venous dilatation and tortuosity in AMS subjects, showing direct correlation with SpO_2_ levels (Gupta et al., 2023). Tinkara et al. examined 19 individuals (11 adults, 8 children) during 20-h hypoxic exposure, observing significant increases in central retinal arteriolar and venular equivalents (Mlinar et al., 2023). Jinlan et al. compared 50 high-altitude and 43 low-altitude residents, reporting lower retinal VDf in the high-altitude group (Ma et al., 2022). Current research primarily focuses on either low-altitude residents during short-term exposure or individuals with altitude illnesses. Limited data exist regarding lifelong hypoxia’s effects on ocular blood flow, particularly retinal vascular changes in high-altitude resident children. While it is generally accepted that a number of changes occur in the cardiovascular system (Archer et al., 2024; Stau et al., 2024), retinal vascular changes under prolonged hypobaric hypoxia remain unclear due to insufficient investigation.

The observation of significantly smaller retinal VC in the HLC group—given that retinal morphology mirrors systemic microvascular health—implies systemic vascular remodeling, an adaptive response to chronic hypobaric hypoxia. Under persistent hypoxic stimulation, individuals may develop hypoxic vasoconstriction, a process mediated by mechanisms such as impaired nitric oxide (NO)-dependent vasodilation, mpaired systemic endothelial function, and hypoxia-induced vascular smooth muscle cell proliferation, which is known to affect vascular in the pulmonary circulation (Bruno et al., 2014; Sheng et al., 2009).

These reduced vessel diameters may reflect both long-term adaptations to high-altitude hypoxia and potential disease associations. Narrowing of retinal arteriole caliber may be associated with increased future blood pressure levels and individuals residing at very high altitudes (3,500–5,800 m) demonstrate a higher prevalence and earlier onset of hypertension and stroke (Sun et al., 2009; Jefferson et al., 2002; Ortiz-Prado et al., 2021). The elevated incidence of cerebrovascular disease among high-altitude populations is associated not only with physiological and environmental factors but also with lifestyle habits, including high-fat diets, smoking, and alcohol consumption (Qi et al., 2020). To reduce cardiovascular risk, adopting a balanced diet from childhood and avoiding smoking and excessive alcohol intake are recommended (Lennon et al., 2021).

Statistical analysis revealed significantly smaller VC in the HLC group compared to the LLC group across all annular zones, however, no significant intergroup differences were observed in VD measurements in this study. Hypoxic exposure has been well-documented to induce various visual impairments, including altered color discrimination (Connolly et al., 2008), impaired dark adaptation (Kobrick and Appleton, 1971), and diminished contrast sensitivity (Pescosolido et al., 2015). Interestingly, despite these known effects, high-altitude adaptation appears to preserve visual acuity in native highland populations (Bosch et al., 2009). This phenomenon may be attributed to the maintenance of retinal vascular homeostasis, as evidenced by the absence of significant VC dilation and preservation of normal VD parameters in high-altitude residents under chronic hypoxic conditions (Stacey et al., 2023).

Comparative analysis demonstrated that both HLC and LLC groups exhibited significantly increased VD in both superior and inferior peripapillary quadrants (p < 0.05). Notably, the HLC group showed a 27.2% higher mean VD value in the inferior quadrant compared to the LLC group (0.098% vs. 0.077%, p < 0.001). This distinct regional vascular distribution pattern, supported by previous studies (Chen and Kardon, 2016; Asrani et al., 2014), suggests that the superior and inferior peripapillary regions may represent anatomical vulnerability zones for compressive damage in various optic neuropathies. The particularly pronounced VD observed in the inferior quadrant of HLC individuals may reflect both compensatory vascular mechanisms and increased susceptibility to optic nerve fiber compression-related pathologies, particularly glaucoma, in this specific region.

A particularly noteworthy finding in our study was the significantly larger optic disc area in the HLC group compared to the LLC group (0.625 mm^2^ vs. 0.557 mm^2^ p < 0.01), despite comparable C/D between the two groups. While the precise mechanism underlying this morphological difference remains unclear, previous research by Ma et al. (2023) demonstrated optic disc and cup enlargement in high-altitude residents with high-altitude polycythemia (HAPC), attributing these changes to hypoxia-induced venous dilatation and impaired axonal transport leading to optic disc edema. However, our study population consisted exclusively of healthy highland children, suggesting a distinct physiological adaptation rather than pathological changes. From a clinical perspective, physiological enlargement of the optic cup typically reflects a larger scleral canal and represents a normal anatomical variation. In contrast, reduced cup size may indicate optic disc crowding, which has been associated with various optic neuropathies, including optic disc drusen (ODD) and non-arteritic anterior ischemic optic neuropathy (NAION) (Sadun and Wang, 2011; Vienne-Jumeau et al., 2023; Singla and Agarwal, 2025). The observed larger optic disc dimensions in HLC individuals may therefore represent an adaptive morphological response to high-altitude conditions, potentially serving as a protective mechanism against optic nerve compression and associated pathologies.

Various hypoxic tolerance patterns observed among different native highlanders, such as Andeans, East African, and Tibetans (Mishra et al., 2015; Simonson, 2015; Cheong et al., 2017). Genetic analysis has revealed marked differences in hypoxic adaptation between Tibetan and Han populations (Yi et al., 2010). This study identified differences in retinal vascular characteristics between high-altitude Tibetan children and their low-altitude Han counterparts, these variations may be attributable to ethnic factors, caution should be exercised in extrapolating these findings to non-Tibetan populations migrating from low to high altitudes.

Although our inclusion criteria—specifying an age of 8–9 years, BCVA ≥20/25, SE ≤ 0.5 D, and AXL <26 mm—aimed to minimize confounders, differences in age and axial length persisted between the two groups. Furthermore, the HLC group had a lower proportion of male participants (43.8%) compared to the LLC group (52.2%). Previous studies have showed that male and greater axial length is associated with finer retinal vessels (He et al., 2024; Lim et al., 2011). Interestingly, however, the HLC group in this study exhibited smaller VC despite having a higher proportion of females and shorter AXL (22.76 mm vs. 23.27 mm). Therefore, the relatively smaller VC observed in the HLC group are more likely attributable to environmental or ethnic factors than to confounding variables such as age, sex, or AXL.

There are some limitations in this study. Firstly, due to the cross-sectional design, there were unable to establish the changes of retinal vascular characteristics in relation to altitude elevation. Secondly, given that the analyses conducted in this study were confined to the scope of fundus photography, there exists a certain bias in accurately representing the complete retinal vasculature. Thirdly, this study had a relatively small sample size, and larger studies are needed to further verify these results. Lastly, a concealed confounding variable in our research and analysis is ethnic diversity. This study compared retinal vascular characteristics between Tibetan children residing at high altitudes and Han Chinese children living at low altitudes. Given that ethnic background factors may have a significant impact on the physiological adaptations to the sustained hypoxia (Cheong et al., 2017; Yi et al., 2010), these findings may not be directly generalizable to other populations worldwide.

This study revealed significant morphological differences in fundus characteristics between the HLC and LLC groups. Specifically, the HLC group demonstrated reduced VC and VBA compared to the LLC group, while exhibiting significantly larger optic disc and cup areas.

## Data Availability

The original contributions presented in the study are included in the article/supplementary material, further inquiries can be directed to the corresponding author.
